# Linker-Dependent Variation in the Photophysical Properties of Dinuclear 2-Phenylpyridinato(salicylaldiminato)platinum(II) Complexes Featuring NDI Units

**DOI:** 10.3390/molecules30122664

**Published:** 2025-06-19

**Authors:** Soichiro Kawamorita, Tatsuya Matsuoka, Kazuki Nakamura, Bijak Riyandi Ahadito, Takeshi Naota

**Affiliations:** Department of Chemistry, Graduate School of Engineering Science, Osaka University, Machikaneyama, Toyonaka 560-8531, Japan; matsuoka@soc.chem.es.osaka-u.ac.jp (T.M.); nakamaura@soc.chem.es.osaka-u.ac.jp (K.N.); bijak@mipa.unsri.ac.id (B.R.A.)

**Keywords:** through-space charge transfer, platinum(II) complex, flexible linker, naphthalene diimide, photophysical properties, intramolecular interactions

## Abstract

Through-space charge transfer (TSCT) between spatially adjacent donor and acceptor units has garnered considerable attention as a promising design principle for optoelectronic materials. While TSCT systems incorporating rigid spacers have been extensively studied to enhance through-space interactions, transition metal complexes connected by flexible linkers remain underexplored, despite increasing interest in their potential TSCT behavior. Herein, we report the design and synthesis of a donor–acceptor–donor (D-A-D)-type complex (**1**), in which a central naphthalenediimide (NDI) electron acceptor is linked to 2-phenylpyridinato(salicylaldiminato)platinum(II) complexes via flexible alkyl linkers. By systematically varying the linker length (*n* = 3, 4, 5, 6; **1a**–**d**), we achieved precise control over the spatial arrangement between the NDI core and the platinum moieties in solution. Notably, compound **1a** (*n* = 3) adopts an S-shaped conformation in solution, giving rise to a distinct TSCT absorption band. The structural and photophysical properties were thoroughly investigated using single-crystal X-ray diffraction, ^1^H NMR, NOESY analysis, and DFT calculations, which collectively support the existence of the folded conformation and associated TSCT behavior. These findings highlight that TSCT can be effectively induced in flexible molecular systems by exploiting intramolecular spatial proximity and non-covalent interactions, thereby offering new avenues for the design of responsive optoelectronic materials.

## 1. Introduction

In recent years, through-space conjugation (TSC), a phenomenon in which aromatic rings are brought into close spatial proximity to enable π-electron delocalization without covalent bonding, has emerged as a promising molecular design strategy for the development of novel optoelectronic materials [1,2,3,4,5,6]. Originally inspired by studies on cyclophanes [7,8,9,10,11,12,13,14,15,16,17,18,19,20,21], TSC has since been extended to a wide range of molecular architectures [22,23,24,25,26,27,28,29,30,31,32,33,34,35,36,37,38,39,40,41], many of which incorporate rigid spacers to enforce the face-to-face alignment of aromatic units. This spatial arrangement facilitates efficient through-space electronic coupling, resulting in red-shifted absorption bands and enhanced charge and energy transfer rates. Among various studies of TSC, through-space charge transfer (TSCT) between spatially adjacent donor and acceptor units has drawn particular attention. Unlike conventional through-bond charge transfer (TBCT), TSCT allows for a significant reduction in the singlet–triplet energy gap ΔE_ST_ while retaining a high transition dipole moment, making it a promising approach for designing thermally activated delayed fluorescence (TADF) materials [42,43,44]. Furthermore, the TSC concept has recently been extended to clusteroluminescence, a photophysical behavior observed in the aggregated state, arising from non-conjugated yet spatially proximate functional groups [45,46,47,48,49]. These advances underscore the growing significance of TSC as a design paradigm for next-generation functional materials.

In phosphorescent transition metal complexes, through-space charge transfer (TSCT) can also be achieved by carefully designing the spatial arrangement between the metal complex and nearby aromatic groups [50,51,52,53,54,55]. In most previous studies, rigid spacers were employed to precisely control the spatial arrangement between donor and acceptor units, thereby enhancing the efficiency of intramolecular interactions. On the other hand, although TSCT involving flexible alkyl linkers has been actively investigated in aggregated states, particularly in the context of clusteroluminogens, reports on TSCT in solution states remain limited [56,57,58,59]. To the best of our knowledge, no studies have reported on transition metal complexes containing acceptor units and connected via flexible linkers, with a focus on TSCT-related photophysical properties in solution. Recently, during the preparation of this submission, an important report was published; although it does not involve flexible linkers, it demonstrates TSCT through the proximity of donor and acceptor units within metal complexes [59].

We have designed a series of three-dimensional transition metal complexes that achieve a balance of conformational flexibility and π-stacking ability by connecting square-planar metal centers via flexible polymethylene or polyethylene glycol (PEG) chains [60,61,62,63,64,65,66,67,68,69,70,71,72,73,74,75,76,77,78,79,80,81,82,83,84]. These molecular architectures have exhibited a wide range of unique properties, including ultrasound-responsive gelation [61,67,84], aggregation-induced emission (AIE) [60,62,63,75,79], chiroptical luminescence [81,82,83], and unique molecular dynamics [64,69,73,76,78].

**Figure 1:**
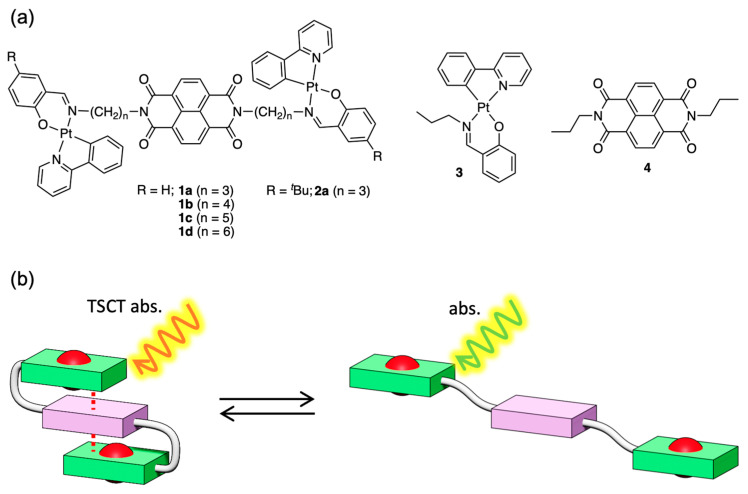
(**a**) Molecular structures of **1a**–**1d**, **2a**, and reference compounds **3** and **4**. (**b**) Schematic illustration of through-space charge transfer (TSCT) absorption depending on the conformational flexibility.

As an extension of this molecular design strategy, herein, we report the design and synthesis of a D–A–D-type complex (**1**), in which a central naphthalenediimide (NDI) electron acceptor is flanked by phenylpyridinato(salicylaldiminato)platinum(II) complexes via flexible alkyl linkers. By systematically varying the linker length (*n* = 3, 4, 5, 6; denoted as **1a**–**d**, Figure 1a), we achieved precise control over the spatial arrangement between the NDI core and the platinum complexes in solution. Notably, in the case of *n* = 3, the molecule adopts an S-shaped folded conformation in solution, in which the platinum complexes are positioned on both sides of the NDI core in close proximity (Figure 1b). This characteristic geometry leads to the emergence of a TSCT (through-space charge transfer) absorption band, which was clearly observed in the UV–vis absorption spectrum. Although our previous work on donor–acceptor systems composed of NDI and electron-rich pyrroleimine units connected by flexible chains did not involve transition metals, it also provided a conceptual foundation for the current study, especially in using conformational folding to modulate intramolecular interactions [85].

## 2. Results

**Figure 2:**
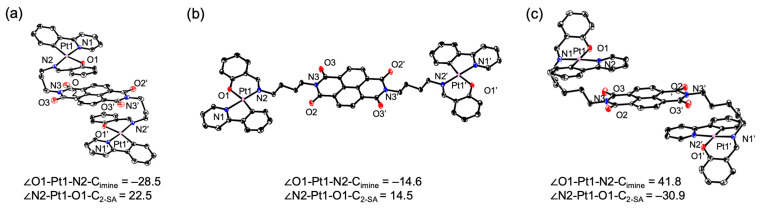
ORTEP representations of (**a**) **1a**, (**b**) **1b**, and (**c**) **1d**. Thermal ellipsoids are shown at the 50% probability level.

**Figure 3:**
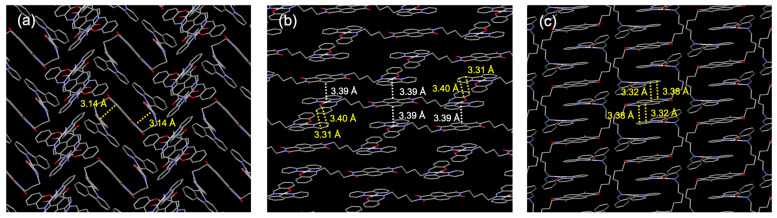
Packing in (**a**) **1a**, (**b**) **1b**, and (**c**) **1d** crystals. The yellow and white dotted lines show C–C and Pt–C interactions, respectively.

The synthesis and purification of the complexes **1a**–**d** and **2** were carried out according to standard procedures, as detailed in the Section 4. Single crystals suitable for X-ray diffraction analysis were obtained by recrystallization from DMSO (**1a**), chloroform/chlorobenzene (**1b**), and dichloromethane/o-dichlorobenzene (**1d**) and subsequently subjected to structural analysis (Figure 2 and Figure 3, Table 1). Crystallographic data for compounds **1a** (CCDC 1850823), **1b** (CCDC 1850824), and **1d** (CCDC 1850825) can be obtained free of charge from the Cambridge Crystallographic Data Centre. All three compounds exhibit a square-planar coordination geometry typical of platinum(II) complexes, in which the pyridyl nitrogen and imine nitrogen atoms adopt a trans configuration. In **1a**, the platinum and NDI units are in close intramolecular contact, forming a folded S-shaped conformation (Figure 2a). In **1b**, the molecule adopts a linear conformation with a fully extended alkyl chain (Figure 2b). In **1d**, the molecular also adopts an S-shaped conformation, but without intramolecular contact between the platinum and NDI units (Figure 2c). An analysis of bond lengths and angles revealed that the five-membered metallacycle formed between the phenylpyridinate ligand and platinum remains highly planar. In contrast, the six-membered chelate ring involving the salicylaldimine moiety exhibits noticeable distortion, deviating from the platinum coordination plane. To quantitatively assess this deviation, two dihedral angles were calculated to describe the out-of-plane displacement of the salicylaldimine unit with respect to the platinum coordination plane: O1–Pt1–N2–C_imine_ and N2–Pt1–O1–C_ipso-O_ (Figure 2). For **1a**, the dihedral angles were −28.5° and 22.5°, respectively, indicating a significant bend. For **1b**, the corresponding values were −14.6° and 14.5°, suggesting a somewhat reduced distortion. In **1d**, the angles were 41.8° and –30.9°, indicating a substantial deviation from planarity.

The stacking structures of each compound are shown in Figure 3. In **1a**, an intramolecular π–π interaction is observed between the NDI unit and the salicylaldimine ring of the Pt unit, with an interplanar distance of 3.14 Å (Figure 3a). This interaction results in a characteristic three-dimensional arrangement in which the platinum complexes are positioned above and below the NDI core. In **1b**, the two linearly aligned Pt units and the central NDI unit engage in intermolecular stacking (Figure 3b). A π–π interaction of 3.39 Å is observed between the platinum complex and the NDI unit, along with interactions of 3.31 Å and 3.40 Å between the salicylaldimine moiety of one complex and the pyridine moiety of an adjacent complex. In **1d**, two dichloromethane molecules are included as crystal solvents (omitted in the figure for clarity, Figure 3c). Although the overall molecular conformation adopts an S-shape, the individual platinum and NDI units form intermolecular stacking interactions. Specifically, π–π interactions of 3.32 Å and 3.38 Å are observed between the pyridine moiety of the Pt complex and the NDI unit of a neighboring molecule.

**Figure 4:**
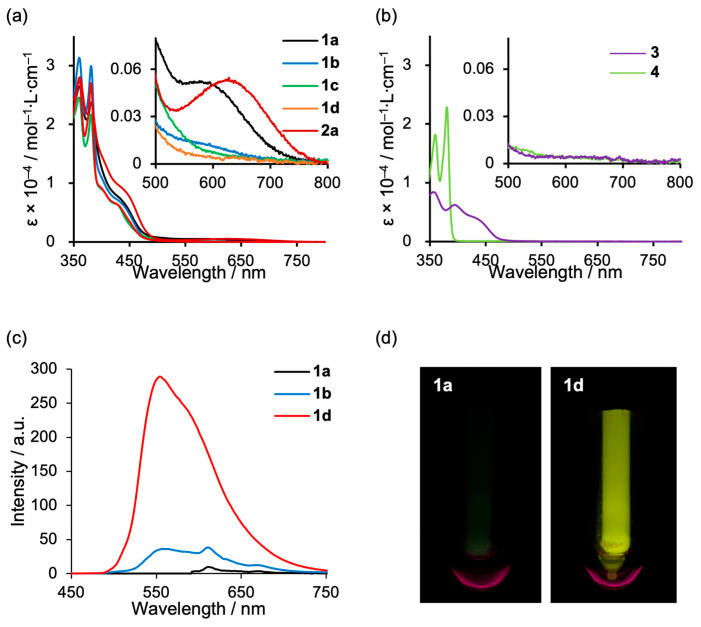
(**a**) UV-vis spectra of complexes **1a**–**1d** and **2a** and (**b**) **3** and **4** in chloroform (1.0 × 10^−4^ M) at 298 K. (**c**) Emission spectra of complexes **1a**, **1b**, and **1d** (λ_ex_ = 430 nm) and (**d**) photos of **1a** and **1d** under UV (365 nm) irradiation in 2-MeTHF (1.0 × 10^−4^ M) at 77 K.

UV–vis absorption spectra were recorded for the D–A–D-type dinuclear platinum complexes **1a**–**d**, the tert-butyl-substituted analogue **2a** (with substitution at the 5-position of the salicylaldimine moiety), the mononuclear platinum complex **3**, and the *N*-alkylated NDI derivative **4** (Figure 4a,b and Table 2). Compounds **1** and **2a** exhibited sharp absorption peaks around 360 and 380 nm, along with broader shoulder-like bands spanning 400–500 nm (Figure 4a). As shown in Figure 4b, these features can be attributed to the individual components—platinum complex **3** and NDI derivative **4**, respectively. The insets of Figure 4a,b show magnified views of the long-wavelength region (600–700 nm) and reveal weak but distinct absorption bands at approximately 600 nm for **1a** and 640 nm for **2a**. A similar, though less intense, band is also observed for **1b**, while no corresponding features are detected for **1c** and **1d**, or the individual compounds **3** and **4**. These results suggest that, in the case of short alkyl linkers (*n* = 3), the NDI and platinum units are brought into close spatial proximity, leading to the emergence of a through-space charge transfer (TSCT) absorption band arising from intramolecular donor–acceptor interactions. In contrast, when the UV-vis spectra were measured in the solid state, a distinct absorption band attributed to intramolecular and intermolecular CT was observed around 600 nm for 1a–d and 2a, while no such features were detected for complexes 3 and 4 (Figure S1).

The emission properties of **1** were further investigated in 2-methyltetrahydrofuran (2-MeTHF) (Figure 4c and Table 1). At room temperature, none of the compounds **1**, **2**, or **3** exhibited any detectable emission. At 77 K, however, compound **1d** (*n* = 6) showed clear phosphorescence, with a quantum yield (*Φ*_77K_) of 0.06. Mononuclear platinum complex **3** was also emissive (*Φ*_77K_ = 0.10, Table 1). In contrast, compound **1a** (*n* = 3), which displayed a TSCT absorption band, showed negligible emission even at 77 K. Similarly, **2a**, which possesses a short alkyl chain, also exhibited no emission. Compound **1b** was also weakly emissive, with a quantum yield of only 0.01. Compound **1c** exhibited poor solubility in 2-MeTHF, making photophysical measurements difficult. The non-emissive behavior observed for the shorter alkyl chain derivatives is likely attributable to the folded S-shaped conformation, which brings the platinum(II) and NDI units into close spatial proximity. This arrangement is presumed to facilitate energy transfer from the triplet excited state of the platinum unit to the triplet state of the NDI unit, followed by nonradiative deactivation, thereby quenching the phosphorescence.

**Figure 5:**
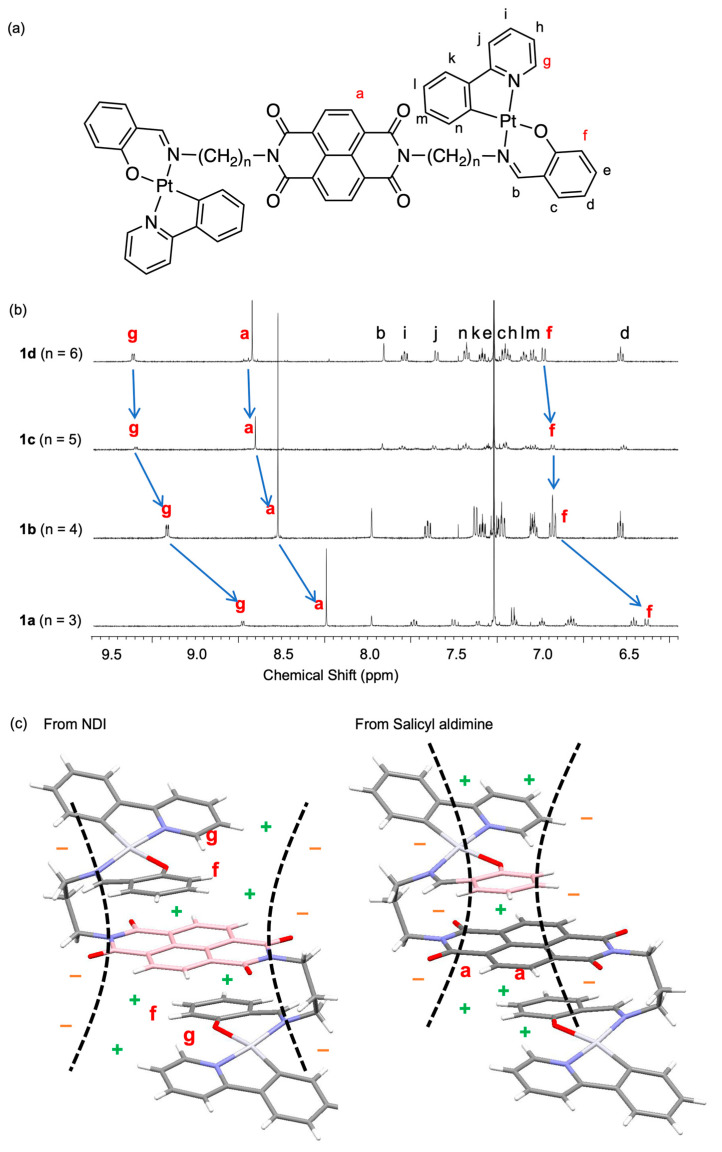
(**a**) Molecular structure of compound **1** with proton assignments. (**b**) ^1^H NMR spectra of compounds **1a**–**d** in CDCl_3_ at room temperature. (**c**) Molecular structure of **1a** visualizing the spatial shielding effects. The left panel highlights shielding from the NDI unit, while the right panel shows shielding from Pt units. The molecular conformation is based on the X-ray crystal structure. The orange (−) and green (+) signs indicate deshielding and shielding effects, respectively.

To investigate the molecular conformations in solution, the ^1^H NMR spectra of compounds **1a**–**d** were measured and analyzed (Figure 5). Particular attention was paid to proton *a* of the NDI moiety, *f* at the 3-position of the salicylaldimine (SA) ring, and *g* at the α-position to nitrogen of the pyridine ring (Figure 5a). As shown in Figure 5b, all three protons exhibited remarkable upfield shifts of up to approximately 0.5 ppm as the spacer-chain length decreased. These chemical shift changes can be rationalized by considering that **1a** adopts a folded S-shaped conformation in solution, consistent with the structure determined by X-ray crystallography. In short-alkyl-chain derivatives, molecular folding brings the aromatic units into close spatial proximity, placing these protons in highly shielded environments. Specifically, as illustrated in Figure 5c (left), protons *g* and *f* are positioned above and below the NDI core, respectively, and are thus subject to shielding by the ring current of the NDI unit. Proton *a*, located between the two SA rings (Figure 5c, right), is similarly shielded by the adjacent aromatic units, resulting in the observed upfield shifts.

**Figure 6:**
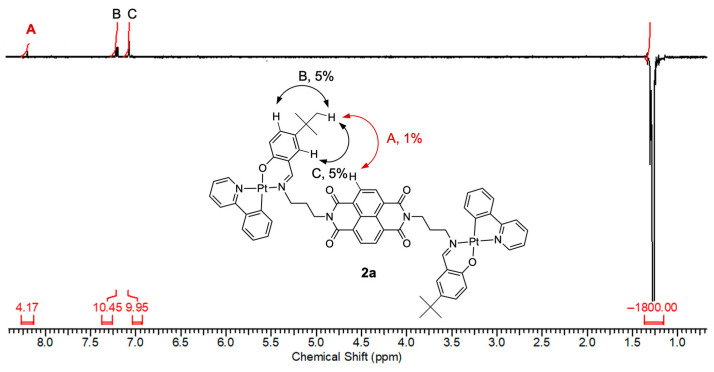
One-dimensional NOESY spectrum of compound **2a** in CDCl_3_. Correlations observed in the spectrum are labeled as A, B, and C. Correlation A, shown in red, indicates the NOE interaction between the *t*-Bu group and the NDI core.

Furthermore, 1D NOESY measurement was performed for **2a** (*n* = 3), which bears *tert*-butyl groups. As shown in Figure 6, an NOE interaction of approximately 1% was detected between the *tert*-butyl groups and proton *a* of the NDI moiety. In a linear conformation, these two groups are spatially distant and would not exhibit NOE. However, in a folded S-shaped conformation, they are brought into close proximity, making the observed NOE interaction possible. These findings are consistent with the TSCT-related absorption band observed for **2a**.

## 3. Discussion

To quantitatively evaluate the thermodynamic stability of the S-shaped conformation observed in **1a**, which influences both its photophysical properties and NMR spectra, DFT calculations were performed to optimize and compare the geometries and energies of both the S-shaped and linear conformers. Geometry optimizations were carried out for the folded S-shaped structure observed in the crystal and for a putative linear conformation, which is assumed to represent an alternative stable form. All calculations were performed using the B3LYP functional with the def2-TZVP basis set for all atoms, and solvent effects were taken into account using the CPCM model with chloroform as the solvent (computational details are provided in Section 4). The results indicated that the S-shaped conformer is more stable, with an energy 25.7 kcal/mol lower than that of the linear conformer.

**Figure 7:**
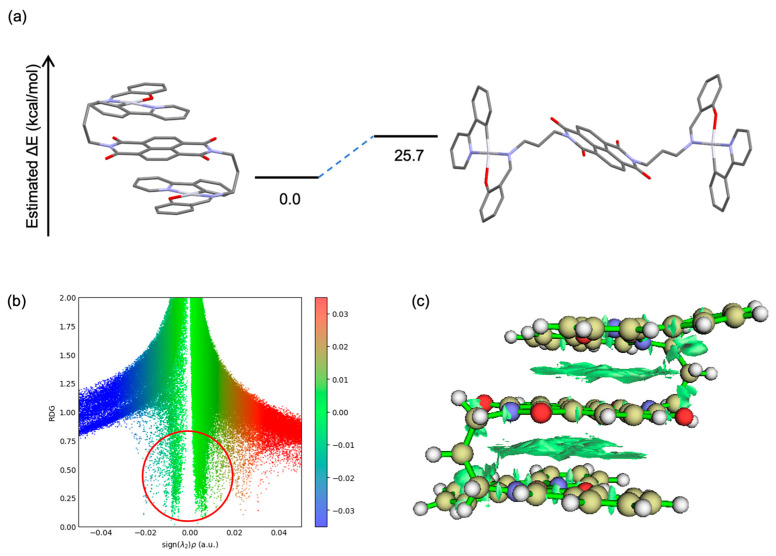
(**a**) Optimized structures and relative energies of compound **1a** in folded (S-shape) and linear (open-shape) conformations. (**b**) Reduced density gradient (RDG) scatter plot of **1a**, showing RDG versus sign(*λ*_2_)*ρ*. The red circle highlights the region where van der Waals or π–π interactions are observed. (**c**) NCI isosurface map of **1a** highlighting non-covalent interactions.

To investigate the interactions responsible for stabilizing the S-shaped geometry, we conducted a non-covalent interaction (NCI) analysis based on the reduced density gradient (RDG) method (Figure 7b). This approach involves plotting the RDG against sign(λ_2_)*ρ*, the product of the electron density (*ρ*) and the sign of the second eigenvalue (λ_2_) of the Hessian matrix, which enables the visualization and classification of weak interactions. Peaks in the RDG near sign(λ_2_)*ρ* ≈ 0 (depicted in green) indicate van der Waals interactions, while negative values (blue) correspond to attractive interactions such as hydrogen bonding, and positive values (red) correspond to steric repulsion. In the RDG scatter plot for **1a** (Figure 7b), a prominent green spike is observed near the center, which is characteristic of van der Waals interactions. In addition, the NCI isosurface plot (Figure 7c) visualizes intramolecular non-covalent interactions as green surfaces, with layered features clearly attributable to π–π stacking. These results support the notion that the folded S-shaped conformation is stabilized by spatially favorable non-covalent interactions.

**Figure 8:**
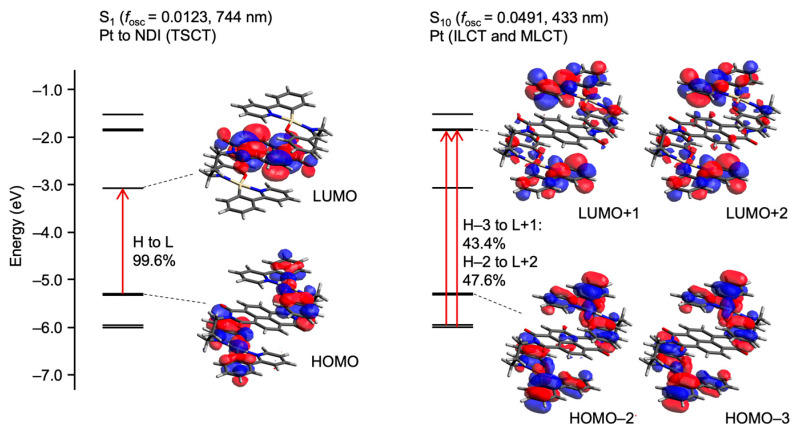
Electronic transitions of compound **1a** estimated by TD-DFT calculations, along with the corresponding oscillator strengths (*f*_osc_) and excitation energies. The **left** panel shows the lowest-energy singlet excited state (S_1_), characterized by a HOMO → LUMO transition. The **right** panel depicts the higher singlet excited state (S_10_), which mainly involves HOMO − 2 → LUMO + 2 and HOMO − 3 → LUMO + 1 transitions.

To gain insight into the nature of the intramolecular TSCT transition, TDDFT calculations were performed for **1a** in its S-shaped conformation (Figure 8). The calculation was based on an optimized geometry from the S-shaped structure obtained by single-crystal X-ray diffraction. The lowest singlet excited state (S_1_) was found to be primarily composed of a HOMO → LUMO transition, with a clear charge transfer character from the platinum complex unit to the NDI moiety. This transition is characterized as a TSCT between spatially adjacent donor and acceptor units. The oscillator strength (*f*_osc_) associated with this transition was 0.0123, indicating a reasonably allowed transition. The calculated excitation wavelength (*λ*_abs_) was 744 nm, which corresponds well with the weak, red-shifted absorption band observed experimentally.

In contrast, a higher-energy excited state (S_10_) exhibited a significantly stronger transition (*f*_osc_ = 0.0491, *λ*_abs_ = 433 nm), primarily consisting of multiple contributions, including HOMO − 2 → LUMO + 2 and HOMO − 3 → LUMO + 1 transitions. These transitions are assigned to metal-to-ligand charge transfer (MLCT) and intra-ligand charge transfer (ILCT) processes within the platinum complex unit. This absorption feature is characteristic of platinum(II) complexes and aligns well with the experimentally observed shoulder band around 442 nm.

## 4. Materials and Methods

General: Melting points were measured on a glass plate on Yanagimoto micro melting point apparatus. IR spectroscopy was performed using a Jasco FT/IR-410 spectrometer. ^1^H and ^13^C NMR spectra were recorded on a Varian Unity–Inova 500 spectrometer (500 MHz for ^1^H, 125 MHz for ^13^C). Chemical shifts are denoted in *δ*-unit (ppm) relative to tetramethylsilane. The splitting patterns are designated as follows: s (singlet), d (doublet), t (triplet), q (quartet), m (multiplet), and br (broad). High-resolution mass spectroscopy (HRMS) was performed using a Bruker microTOF II-OCU spectrometer. UV-vis spectra were obtained using a Jasco V650 spectrometer (Jasco International Co., Ltd., Tokyo, Japan). Emission spectra were acquired with a Jasco FP-6500 spectrometer (Jasco International Co., Ltd., Tokyo, Japan). Absolute quantum yields were determined using a Jasco FP-6500 spectrometer equipped with a Jasco ISN-470 (Jasco International Co., Ltd., Tokyo, Japan) integrating sphere. Compounds **3** [86] and **4** [87] were prepared following the reported method.

X-ray Structure Determination: Crystals employed for X-ray diffraction studies were obtained by recrystallization from DMSO solution for **1a**, chloroform/chlorobenzene solution for **1b,** and dichloromethane/*o*-dichlorobenzene for **1d**, and data were collected using a XtaLAB P-200 diffractometer (Rigaku Corporation, Tokyo, Japan) with graphite-monochromated Mo Kα radiation (λ = 0.71075 Å). Their structures were solved by direct methods and refined using the full-matrix least-squares method. In the subsequent refinement, the function ∑ω(*F*o^2^ − *F*c^2^)^2^ was minimized, where *F*o and *F*c are the observed and calculated structure factor amplitudes, respectively. The positions of non-hydrogen atoms were determined from difference Fourier electron-density maps and refined anisotropically. All the ORTEP illustrations were generated using ORTEP-3.

Computational Methods: All calculations were performed using the ORCA 6.0.0 program package [88]. Geometry optimizations and vibrational frequency analyses of complex **1a**, including both the S-shaped and open-shaped conformers, were carried out using density functional theory (DFT) with the B3LYP functional [89]. The def2-TZVP basis set [90] was employed for all atoms. To reduce computational cost, the RIJCOSX approximation was applied in conjunction with the def2/J auxiliary basis set. Solvent effects were taken into account using the CPCM model with chloroform as the solvent. Analytical frequency calculations were performed at the same level of theory to confirm that the optimized structures correspond to true minima and to obtain zero-point vibrational energy (ZPVE) and thermochemical corrections. Time-dependent DFT (TDDFT) calculations were also conducted at the same level of theory to evaluate the electronic excitation energies and oscillator strengths of the optimized S-shaped conformer.

Synthesis of NDI-Based Ligands: The ligands were synthesized according to the general scheme shown below. A solution of 1,ω-alkanediamine (*n* = 3, 4, 5, or 6) in 1,4-dioxane was treated dropwise with a 1,4-dioxane solution of di-*tert*-butyl dicarbonate at 0 °C. The mixture was stirred at room temperature for 22 h to afford the mono-Boc-protected diamine as a pale-yellow oil. The product was used in the subsequent step without further purification. A mixture of naphthalene-1,4,5,8-tetracarboxylic dianhydride (NDA), the mono-Boc-protected diamine, and triethylamine was heated under reflux at 100 °C for 72 h to afford the corresponding *N*-substituted naphthalenediimide (NDI) derivative. The product was used directly in the next step without further purification. The Boc protecting groups on both terminal alkylamines were removed by treatment with trifluoroacetic acid (TFA) at room temperature for 2 h. A reddish-purple solid was obtained in quantitative yield and used in the subsequent reaction without purification. The resulting diamine-functionalized NDI derivative was reacted with salicylaldehyde in methanol in the presence of sodium carbonate. The mixture was stirred at room temperature for 20 h, and the resulting yellow solid was obtained by reprecipitation.


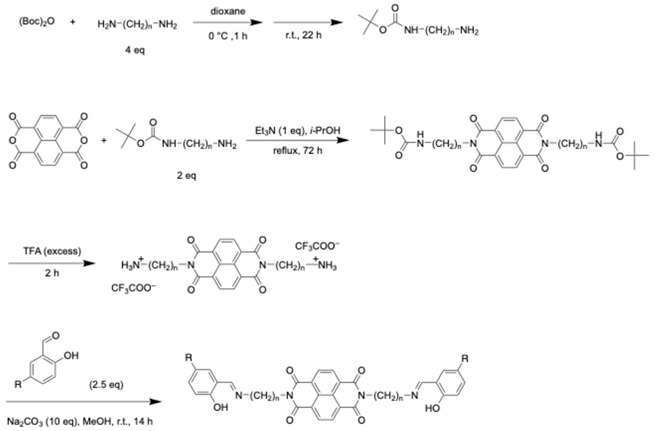


Synthesis of Complexes **1** and **2**: The synthesis of platinum complexes **1a**–**d** and **2a** was exemplified by the preparation of complex **1a**. A mixture of chloro-bridged 2-phenylpyridine platinum(II) dimer (106.9 mg, 0.18 mmol) and the corresponding ligand (140.7 mg, 0.18 mmol) was suspended in a 1:1 mixture of dimethyl sulfoxide and toluene (total volume: 200 mL). To this suspension, K_2_CO_3_ (304.2 mg, 2.20 mmol) was added, and the resulting mixture was stirred at 120 °C for 36 h. After cooling to room temperature, the reaction mixture was extracted with dichloromethane, and the combined organic layers were dried over anhydrous MgSO_4_. The solvent was removed under reduced pressure, and the crude product was purified by silica gel column chromatography to afford complex **1a** as a green solid (51.6 mg, 22%).

**1a** (*n* = 3): Isolated yield: 22%; M.p. = 209–210 °C; IR (KBr): 3047, 2955, 2924, 2854, 1700, 1662, 1607, 1582, 1537, 1486, 1451, 1338, 755, 732 cm^−1^; ^1^H NMR (500 MHz, CDCl_3_) δ 2.51 (tt, *J* = 6.2, 6.7 Hz, 4 H, CH_2_C*H*_2_CH_2_), 4.33 (t, *J* = 6.2 Hz, 4 H, C = NC*H*_2_), 4.44 (t, *J* = 6.7 Hz, 4 H, CONC*H*_2_), 6.39 (d, *J* = 8.7 Hz, 2 H, H^f^), 6.45 (ddd, *J* = 7.8, 6.5, 1.1 Hz, 2 H, H^d^), 6.80 (ddd, *J* = 7.5, 7.3, 1.1 Hz, 2 H, H^l^), 6.83 (ddd, *J* = 7.4, 7.3, 1.4 Hz, 2 H, H^m^), 6.97 (ddd, *J* = 7.5, 5.9, 1.1 Hz, 2 H, H^h^), 7.14 (dd, *J* = 6.5, 1.7 Hz, 2 H, H^c^), 7.15 (ddd, *J* = 8.7, 7.8, 1.7 Hz, 2 H, H^e^), 7.25 (dd, *J* = 7.5, 1.4 Hz, 2 H, H^a^), 7.33 (dd, *J* = 7.4, 1.1 Hz, 2 H, H^k^), 7.47 (dd, *J* = 8.0, 1.1 Hz, 2 H, H^n^), 7.70 (ddd, *J* = 8.0, 7.5, 0.9 Hz, 2 H, H^i^), 7.96 (s, 2 H, H^b^), 8.19 (s, 4 H, H^a^), 8.71 (dd, *J* = 5.9, 0.9 Hz, 2 H, H^g^); ^13^C NMR: not recorded due to the compound’s low solubility; HRMS (ESI^+^): *m*/*z* calcd for C_56_H_43_N_6_O_6_^195^Pt_2_: 1285.2520; found: 1285.2513 [M + H]^+^.

**1b** (*n* = 4): Isolated yield: 18%; M.p. = 284–285 °C; IR (KBr): 3046, 2955, 2934, 2864, 1702, 1662, 1607, 1583, 1539, 1488, 1473, 1455, 1340, 752, 731 cm^−1^; ^1^H NMR (500 MHz, CDCl_3_) δ 1.87 (tt, *J* = 7.2, 7.2 Hz, 4 H), 2.05 (tt, *J* = 7.2, 7.2 Hz, 4 H), 4.21 (t, *J* = 7.2 Hz, 4 H), 4.38 (t, *J* = 7.2 Hz, 4 H), 6.53 (ddd, *J* = 7.8, 6.8, 1.1 Hz, 2 H, H^d^), 6.91 (dd, *J* = 8.5, 1.1 Hz, 2 H, H^f^), 6.92 (ddd, *J* = 8.0, 7.4, 1.1 Hz, 2 H, H^m^), 7.03 (ddd, *J* = 7.4, 6.2, 1.4 Hz, 2 H, H^l^), 7.04 (ddd, 7.4, 6.2, 1.4 Hz, 2 H, H^h^), 7.21 (dd, *J* = 8.0, 1.4 Hz, 2 H, H^n^), 7.23 (dd, *J* = 7.8, 1.8 Hz, 2 H, H^c^), 7.33 (ddd, *J* = 8.5, 6.8, 1.8 Hz, 2 H, H^e^), 7.37 (d, *J* = 8.0, 1.4 Hz, 2 H, H^j^), 7.37 (d, *J* = 6.2, 1.1 Hz, 2 H, H^k^), 7.64 (ddd, *J* = 8.0, 7.4, 1.6 Hz, 2 H, H^i^), 7.97 (s, 2 H, H^b^), 8.51 (s, 4 H, H^a^), 9.15 (dd *J* = 6.2, 1.6 Hz, 2 H, H^g^); ^13^C NMR: not recorded due to the compound’s low solubility; HRMS (APCI): *m*/*z* calcd for C_58_H_47_N_6_O_6_^195^Pt_2_: 1313.2845; found: 1313.2809 [M + H]^+^.

**1c** (*n* = 5): Isolated yield: 11%; M.p. = 197–198 °C; IR (KBr): 3064, 2962, 2931, 2858, 1701, 1661, 1608, 1583, 1535, 1484, 1448, 1342, 760, 737 cm^−1^; ^1^H NMR (500 MHz,CDCl_3_) δ 1.48 (tt, *J* = 7.2, 7.2 Hz, 4 H), 1.71 (tt, *J* = 7.2, 7.2 Hz, 4 H), 1.97 (tt, *J* = 7.2 Hz, 4 H), 4.10 (t, *J* = 7.2 Hz, 4 H), 4.32 (t, *J* = 7.2 Hz, 4 H), 6.51 (dd, *J* = 8.0, 7.3 Hz, 2 H, H^d^), 6.92 (d, *J* = 8.0 Hz, 2 H, H^f^), 7.01 (dd, *J* = 7.6, 7.3 Hz, 2 H, H^m^), 7.06 (ddd, *J* = 7.8, 7.6 1.6 Hz, 2 H, H^l^), 7.17 (dd, 6.2, 6.2 Hz, 2 H, H^h^), 7.20 (d, *J* = 8.0, 1.9 Hz, 2 H, H^c^), 7.31 (dd, *J* = 8.0, 7.3, 1.9 Hz, 2 H, H^e^), 7.39 (d, *J* = 7.8 Hz, 2 H, H^k^), 7.41 (d, *J* = 7.3, 1.6 Hz, 2 H, H^n^), 7.58 (d, *J* = 8.0 Hz, 2 H, H^j^), 7.78 (dd, *J* = 8.0, 6.2 Hz, 2 H, H^i^), 7.90 (s, 2 H, H^b^), 8.58 (s, 4 H, H^a^), 9.30 (d, *J* = 6.2 Hz, 2 H, H^g^); ^13^C NMR: not recorded due to the compound’s low solubility.

**1d** (*n* = 6): Isolated yield: 16%; M.p. = 155–156 °C; IR (KBr): 3049, 3014, 2933, 2858, 1701, 1663, 1608, 1583, 1536, 1484, 1467, 1453, 1339, 755, 732 cm^−1^; ^1^H NMR (500 MHz,CDCl_3_) δ 1.40 (m, 8 H),1.67 (quin, *J* = 7.4 Hz, 4 H), 1.91 (quin, *J* = 7.2 Hz, 4 H), 4.11 (t, *J* = 7.4 Hz, 4 H), 4.31 (t, *J* = 7.3 Hz, 4 H), 6.53 (ddd, *J* = 8.0, 6.9, 0.8 Hz, 2 H, H^d^), 6.98 (dd, *J* = 8.6, 0.8 Hz, 2 H, H^f^), 7.04 (ddd, *J* = 7.8, 7.2, 1.1 Hz, 2 H, H^m^), 7.09 (ddd, *J* = 7.8, 7.2, 1.6 Hz, 2 H, H^l^), 7.19 (ddd, 7.3, 5.8, 1.2 Hz, 2 H, H^h^), 7.21 (dd, *J* = 8.0, 1.9 Hz, 2 H, H^c^), 7.34 (ddd, *J* = 8.6, 6.9, 1.9 Hz, 2 H, H^e^), 7.41 (dd, *J* = 7.8, 1.1 Hz, 2 H, H^k^), 7.43 (dd, *J* = 7.8, 1.6 Hz, 2 H, H^n^), 7.59 (dd, *J* = 8.0, 1.2 Hz, 2 H, H^j^), 7.78 (ddd, *J* = 8.0, 7.3, 1.5 Hz, 2 H, H^i^), 7.90 (s, 2 H, H^b^), 8.66 (s, 4 H, H^a^), 9.34 (dd, *J* = 5.8, 1.5 Hz, 2 H, H^g^); ^13^C NMR: not recorded due to the compound’s low solubility; HRMS (APCI): *m*/*z* calcd for C_62_H_54_N_6_O_6_^195^Pt_2_: 1368.3393; found: 1368.3402 [M]^+^.

**2a** (*n* = 3): Isolated yield: 30%; M.p. = 232–233 °C; IR (KBr) 3047, 2958, 2900, 2865, 1702, 1664, 1617, 1584, 1530, 1478, 1456, 1334, 755, 731 cm^−1^; ^1^H NMR (500 MHz, CDCl_3_) δ 1.26 (s, 18 H), 2.51 (tt, *J* = 6.3 Hz, 4 H), 4.33 (t, *J* = 6.3 Hz, 2 H), 4.44 (t, *J* = 6.3 Hz, 2 H), 6.32 (d, *J* = 8.8 Hz, 2 H, H^f^), 6.78 (ddd, *J* = 7.5, 7.0, 1.2 Hz, 2 H, H^m^), 6.83 (ddd, *J* = 7.6, 7.0, 1.6 Hz, 2 H, H^l^), 6.99 (ddd, *J* = 7.3, 5.9, 1.2 Hz, 2 H, H^h^), 7.07 (d, *J* = 2.7 Hz, 2 H, H^c^), 7.21 (dd, *J* = 8.8, 2.7 Hz, 2 H, H^e^), 7.23 (dd, *J* = 7.6, 1.2 Hz, 2 H, H^k^), 7.33 (dd, *J* = 7.5, 1.6 Hz, 2 H, H^n^), 7.46 (dd, *J* = 7.8, 1.2 Hz, 2 H, H^j^), 7.71 (ddd, *J* = 7.8, 7.3, 1.5 Hz, 2 H, H^i^), 8.18 (s, 2 H, H^b^), 8.26 (s, 4 H, H^a^), 8.76 (dd, *J* = 5.9, 1.5 Hz, 2 H, H^g^); ^13^C NMR (126 MHz, CDCl_3_): 29.7, 31.4, 32.0, 38.8, 63.5, 117.9, 120.4, 121.15, 121.20, 122.6, 123.2, 126.2, 126.6, 128.7, 129.2, 130.1, 132.9, 134.2, 137.3, 138.2, 139.6, 145.7, 145.9, 162.9, 163.2, 164.1, 167.3.; HRMS (APCI): *m*/*z* calcd for C_64_H_58_N_6_O_6_^195^Pt_2_: 1396.3706; found: 13,963,696 [M] ^+^.

**3**: Isolated yield: 72%; M.p. = 220–221 °C; IR (KBr) 3055, 2964, 2931, 2863, 1603, 1441, 1315, 1140, 759, 594 cm^−1^; ^1^H NMR (500 MHz, CDCl_3_) δ 0.93 (t, *J* = 7.4 Hz, 3 H), 1.93 (tt, *J* = 7.4, 7.4 Hz, 2 H), 4.29 (t, *J* = 7.4 Hz, 2 H), 6.54 (td, *J* = 7.4, 1.1 Hz, 1 H, H^d^), 7.00 (d, *J* = 8.6 Hz, 1 H, H^f^), 7.07 (td, *J* = 7.4, 1.1 Hz, 1 H, H^m^), 7.12 (td, *J* = 7.6, 1.7 Hz, 1 H, H^l^), 7.19–7.24 (m, 2 H, H^h and c^), 7.34 (ddd, *J* = 8.6, 7.2, 1.7 Hz, 1 H, H^e^), 7.45 (dd, *J* = 7.6, 1.1 Hz, 1 H, H^k^), 7.48 (dd, *J* = 7.4, 1.7 Hz, 1 H, H^n^), 7.64 (d, *J* = 8.0 Hz, 1 H, H^j^), 7.81 (td, *J* = 8.0, 1.7 Hz, 1 H, H^i^), 7.90 (s, 1 H, H^b^), 9.39 (dd, *J* = 5.4, 1.7 Hz, 1 H, H^g^); ^13^C NMR: 11.1, 27.4, 67.0, 115.0, 118.1, 120.8, 121.9, 122.7, 122.9, 123.4, 129.3, 133.4, 134.6, 134.8, 138.5, 139.4, 146.0, 146.4, 162.3, 166.5, 167.4; HRMS (APCI): *m*/*z* calcd for C_21_H_20_N_2_O^195^Pt: 512.1298; found: 512.1311 [M + H]^+^; Anal. Calcd for C_21_H_20_N_2_OPt: C, 49.31; H, 3.94; N, 5.48. Found: C, 49.29; H, 3.59; N, 5.43.

**4**: Isolated yield: 52%; M.p. = 280–281 °C; IR (KBr) 3095, 2965, 2938, 2873, 1707, 1661, 1579, 1338, 1241, 1074, 885, 765 cm^−1^; ^1^H NMR (500 MHz, CDCl_3_) δ 1.02 (t, *J* = 7.4 Hz, 6 H), 1.77 (tt, *J* = 7.4, 7.4 Hz, 4 H), 4.16 (t, *J* = 7.4 Hz, 4 H), 8.75 (s, 4 H, H^a^); ^13^C NMR: 11.5, 21.4, 42.4, 126.62, 126.69, 130.9, 162.9; HRMS (APCI): *m*/*z* calcd for C_20_H_19_N_2_O_4_: 351.1339; found: 351.1313 [M + H]^+^; Anal. Calcd for C_20_H_18_N_2_O_4_: C, 68.56; H, 5.18; N, 8.00. Found: C, 68.37; H, 4.96; N, 8.09.

## 5. Conclusions

In this study, we designed and synthesized a series of D–A–D-type Pt–NDI–Pt molecules, in which a transition metal donor unit 2-phenylpyridinato(salicylaldiminato)platinum(II) and an electron-accepting NDI unit are connected via flexible alkyl chains. Their structural features and photophysical behaviors were comprehensively examined to evaluate the emergence of TSCT. Single-crystal X-ray diffraction revealed that, for **1a** with an alkyl chain length of *n* = 3, the platinum complexes and the NDI core adopt a folded S-shaped geometry with close intramolecular spatial proximity. Correspondingly, a new long-wavelength absorption band, attributed to TSCT, was observed. In contrast, photoluminescence measurements at low temperature showed that emission was significantly suppressed in the short-chain TSCT-active compound, while the longer-chain analogue exhibited clear phosphorescence. This suggests that nonradiative deactivation via triplet energy transfer to the NDI unit occurs in the TSCT geometry, inhibiting emission. ^1^H NMR and NOESY analyses indicated that the folded conformation is dominant even in solution, and DFT calculations confirmed that the S-shaped structure is thermodynamically more stable than the linear one. These findings collectively demonstrate that TSCT behavior can be clearly induced in flexible molecular systems by leveraging intramolecular spatial arrangement and non-covalent interactions. While TSCT offers an effective strategy for tuning absorption properties, it may adversely affect emissive behavior in certain donor–acceptor systems, as exemplified by the present Pt–NDI combination, revealing a functional trade-off in TSCT-based molecular design.

Future directions include the introduction of additional interactions such as hydrogen bonding or CH–π interactions to further control molecular conformation and simultaneously enhance both TSCT and emission efficiency. Moreover, dynamic control of the TSCT state through external stimuli may broaden the applicability of the present design concept to responsive luminescent materials.

## Figures and Tables

**Table 1:** Crystallographic data for **1a**, **1b**, and **1d**.

	1a	1b	1d
formula	C_56_H_42_N_6_O_6_Pt_2_	C_58_H_46_N_6_O_6_Pt_2_	C_64_H_58_Cl_4_N_6_O_6_Pt_2_
*M*_r_	1285.16	1313.22	1539.19
*T*/K	113	113	113
crystal color, habit	gray, block	green, block	Green, chip
crystal size/mm	0.01 × 0.07 × 0.02	0.04 × 0.03 × 0.01	0.08 × 0.05 × 0.01
crystal system	monoclinic	monoclinic	triclinic
space group	*P*2_1_/c (#14)	*P*2_1_/n (#14)	*P*-$\bar{1}$ (#2)
*a*/Å	12.333 (2)	12.1907 (14)	11.075 (2)
*b*/Å	17.667 (3)	16.0374 (18)	11.224 (3)
*c*/Å	10.1396 (16)	11.8028 (14)	13.138 (3)
*α*/°	90	90	105.686 (3)
*β*/°	96.805 (4)	93.090 (3)	92.943 (2)
*γ*/°	90	90	113.786 (3)
*V*/Å^3^	2193.8 (6)	2304.2 (5)	1414.7 (6)
*Z*	2	2	1
*ρ*_calcd_/g·cm^−3^	1.945	1.893	1.807
*μ* (Mo_Kα_)/cm^−1^	64.091	61.044	51.680
*F*(000)	1248.00	1280.00	756.00
2*θ*_max_/°	54.9	55.0	55.1
No. of reflns measd	21,424	33,122	27,337
No. of obsd reflns	5000	5280	6502
No. variables	316	325	370
*R*_1_ (*I* > 2*σ*(*I*)) ^a^	0.0281	0.0283	0.0446
*wR*_2_ (all reflns) ^b^	0.0616	0.0533	0.0662
Goodness of fit	0.960	1.027	1.022

^a^ *R*_1_ = Σ(|*F*_o_| − |*F*_c_|)/Σ(|*F*_o_|), where *F*_o_ and *F*_c_ are the observed and calculated structure factor amplitudes, respectively. ^b^ *wR*_2_ = [Σ[w(*F*_o_^2^ − *F*_c_^2^)^2^]/Σw(*F*_o_^2^)^2^]^1/2^.

**Table 2:** Photophysical data for **1–4**.

Complex	*λ*_abs_ [nm] ^a^	*λ*_em_ [nm] ^b^	*Φ_77K_* ^b,c^
**1a**	363, 382, 442 (sh), 600	-	-
**1b**	360, 381, 442 (sh)	579	0.01
**1c**	360, 381, 436 (sh)	-	-
**1d**	360, 381, 429 (sh)	554	0.06
**2a**	361, 382, 451 (sh), 628	-	-
**3**	444 (sh)	551	0.10
**4**	360, 380	-	-

^a^ Data were obtained as chloroform solution (1.0 × 10^−4^ M) at 298 K. ^b^ Data were obtained at 77 K under excitation at 430 nm. ^c^ Determined by the absolute method using an integrating sphere.

## Data Availability

The original contributions presented in this study are included in the article. Further inquiries can be directed to the corresponding authors.

## Supplementary Materials

The following supporting information can be downloaded at https://www.mdpi.com/article/10.3390/molecules30122664/s1: Figure S1: UV-vis spectra of (a) complexes **1a**–**1d** and **2a** and (b) **3** and **4** in the solid state at 298 K; Figure S2: ^1^H NMR spectra (500 MHz) of **1a** in CDCl_3_; Figure S3: ^1^H NMR spectra (500 MHz) of **1b** in CDCl_3_; Figure S4: ^1^H NMR spectra (500 MHz) of **1c** in CDCl_3_; Figure S5: ^1^H NMR spectra (500 MHz) of **1d** in CDCl_3_; Figure S6: ^1^H NMR spectra (500 MHz) of **2a** in CDCl_3_; Figure S7: ^13^C NMR spectra (125 MHz) of **2a** in CDCl_3_; Figure S8: ^1^H NMR spectra (500 MHz) of **3** in CDCl_3_; Figure S9: ^13^C NMR spectra (125 MHz) of **3** in CDCl_3_; Figure S10: ^1^H NMR spectra (500 MHz) of **4** in CDCl_3_; Figure S11: ^13^C NMR spectra (125 MHz) of **4** in CDCl_3_.
