# Supplementary material for: Linker-Dependent Variation in the Photophysical Properties of Dinuclear 2-Phenylpyridinato(salicylaldiminato)platinum(II) Complexes Featuring NDI Units
Source: Molecules. 2025 Jun 19;30(12):2664. doi: 10.3390/molecules30122664 (PMC12195916; doi:10.3390/molecules30122664)
Supplement: Supplementary file 1 [file molecules-30-02664-s001.zip › molecules-3677199-supplementary.pdf]

Contents

Figure S1. UV-vis spectra of complexes (a) **1a**–**1d** and **2a**, and (b) **3** and **4** in the solid state at 298 K. 2

Figure S2. ^{1}H NMR spectra (500 MHz) of **1a** in $CDCl_3$. 3

Figure S3. ^{1}H NMR spectra (500 MHz) of **1b** in $CDCl_3$. 3

Figure S4. ^{1}H NMR spectra (500 MHz) of **1c** in $CDCl_3$. 4

Figure S5. ^{1}H NMR spectra (500 MHz) of **1d** in $CDCl_3$. 4

Figure S6. ^{1}H NMR spectra (500 MHz) of **2a** in $CDCl_3$. 5

Figure S7. ^{13}C NMR spectra (125 MHz) of **2a** in $CDCl_3$. 5

Figure S8. ^{1}H NMR spectra (500 MHz) of **3** in $CDCl_3$. 6

Figure S9. ^{13}C NMR spectra (125 MHz) of **3** in $CDCl_3$. 6

Figure S10. ^{1}H NMR spectra (500 MHz) of **4** in $CDCl_3$. 7

Figure S11. ^{13}C NMR spectra (125 MHz) of **4** in $CDCl_3$. 7

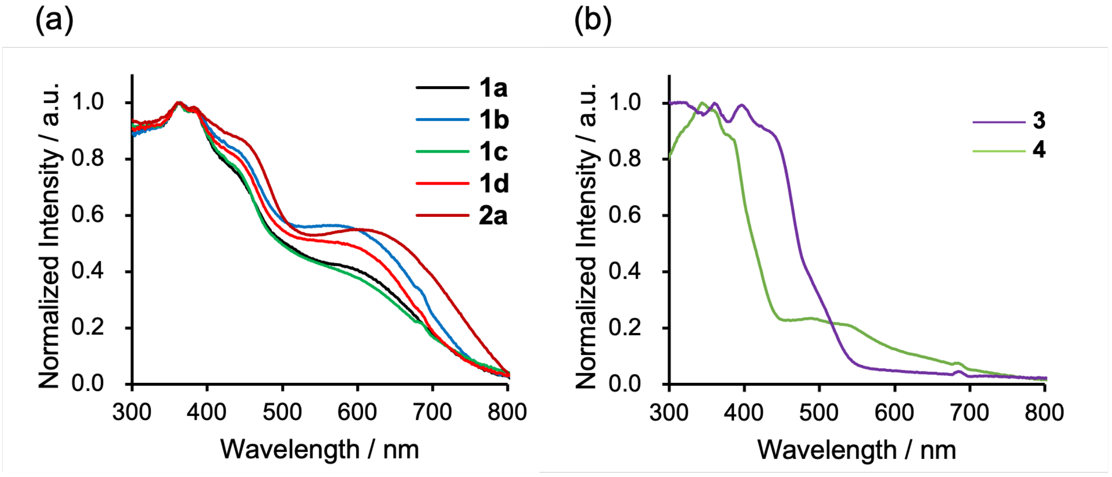

Figure S1. UV-vis spectra of complexes (a) **1a**–**1d** and **2a**, and (b) **3** and **4** in the solid state at 298 K.

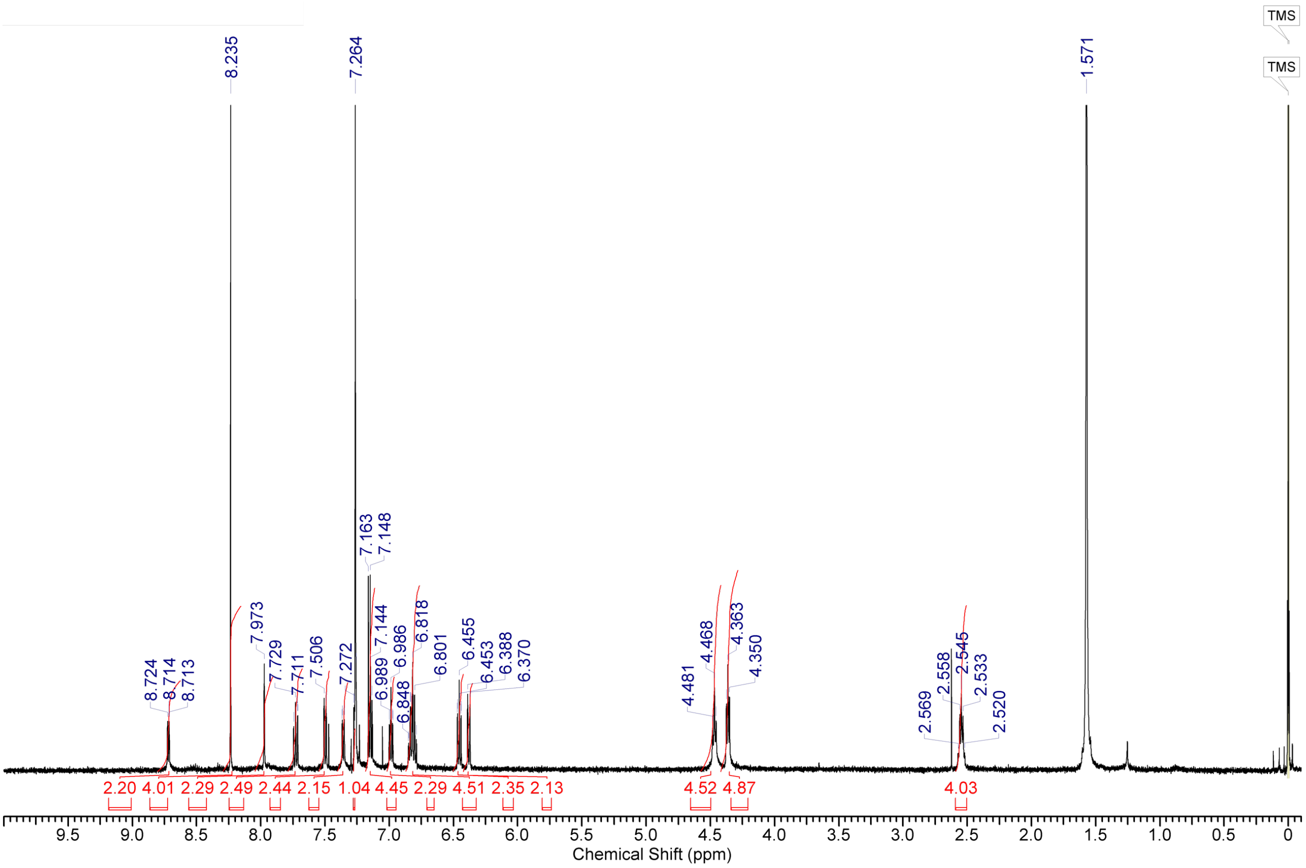

Figure S2. ^{1}H NMR spectra (500 MHz) of **1a** in $CDCl_3$.

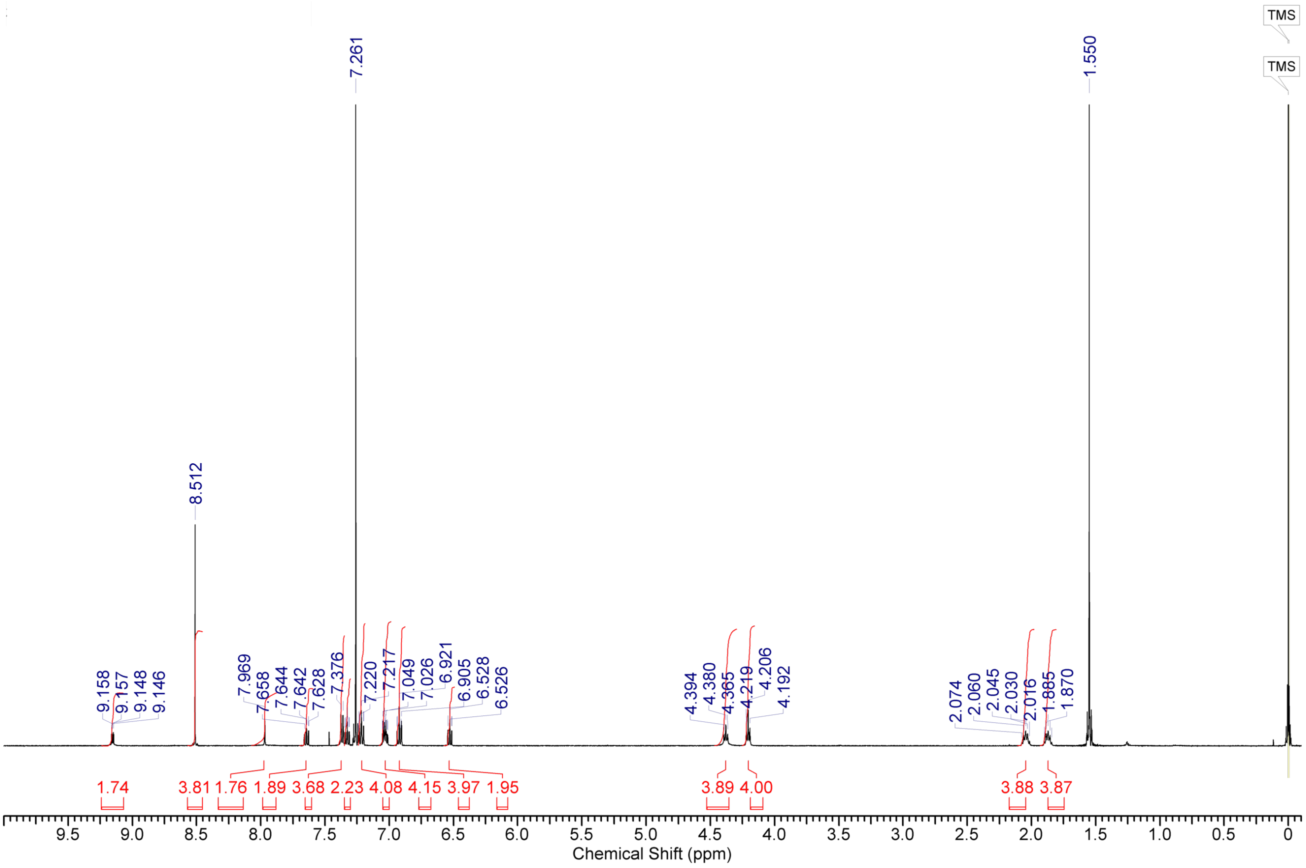

Figure S3. ^{1}H NMR spectra (500 MHz) of **1b** in $CDCl_3$.

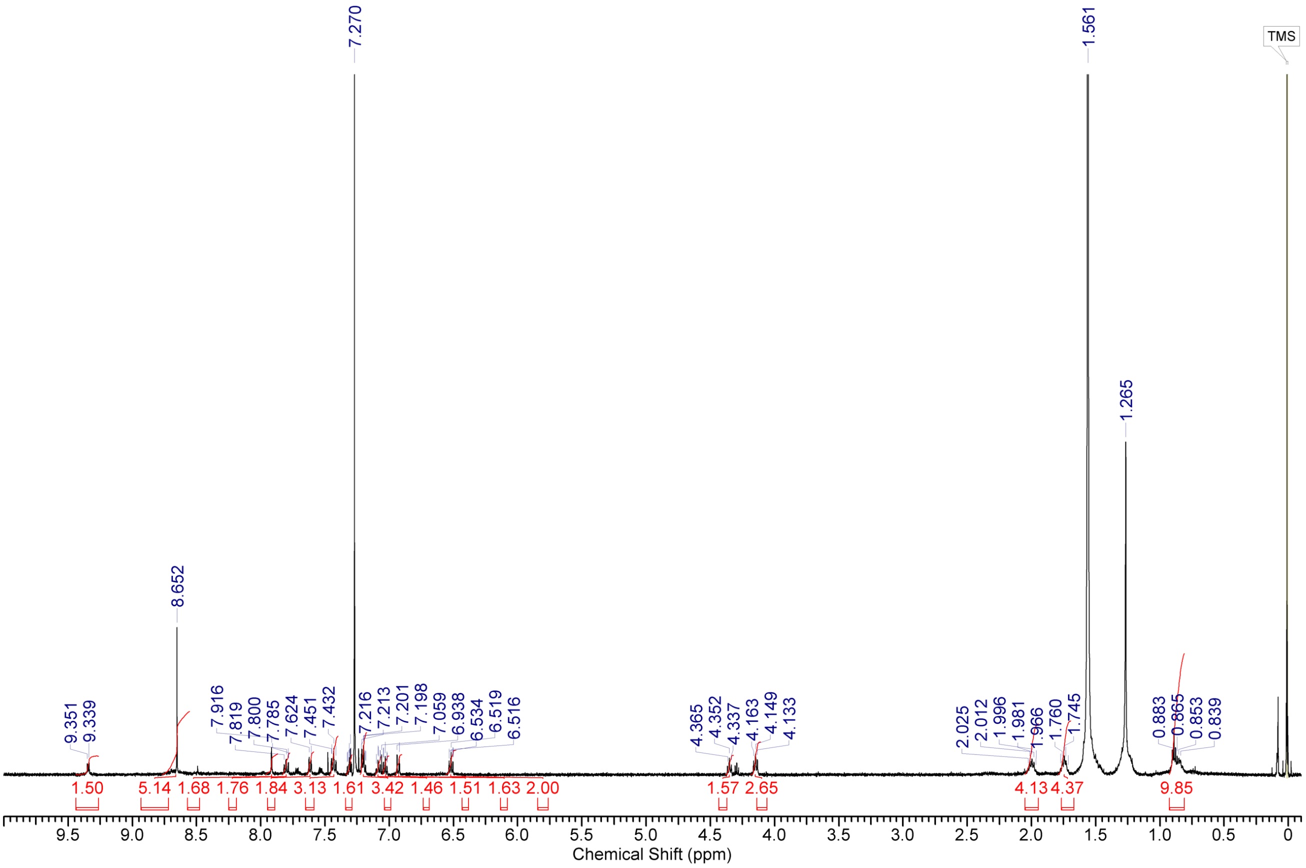

Figure S4. ^{1}H NMR spectra (500 MHz) of **1c** in $CDCl_3$.

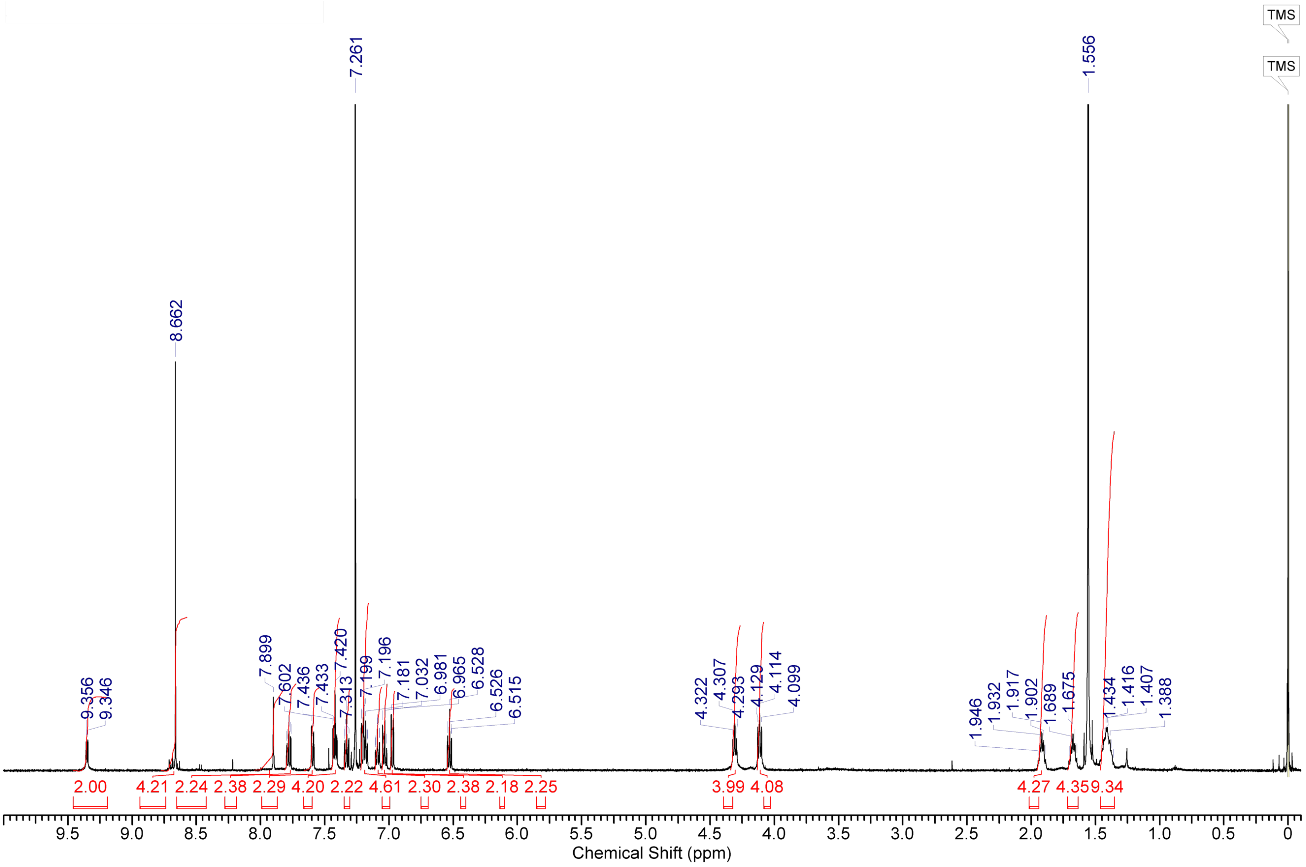

Figure S5. ^{1}H NMR spectra (500 MHz) of **1d** in $CDCl_3$.

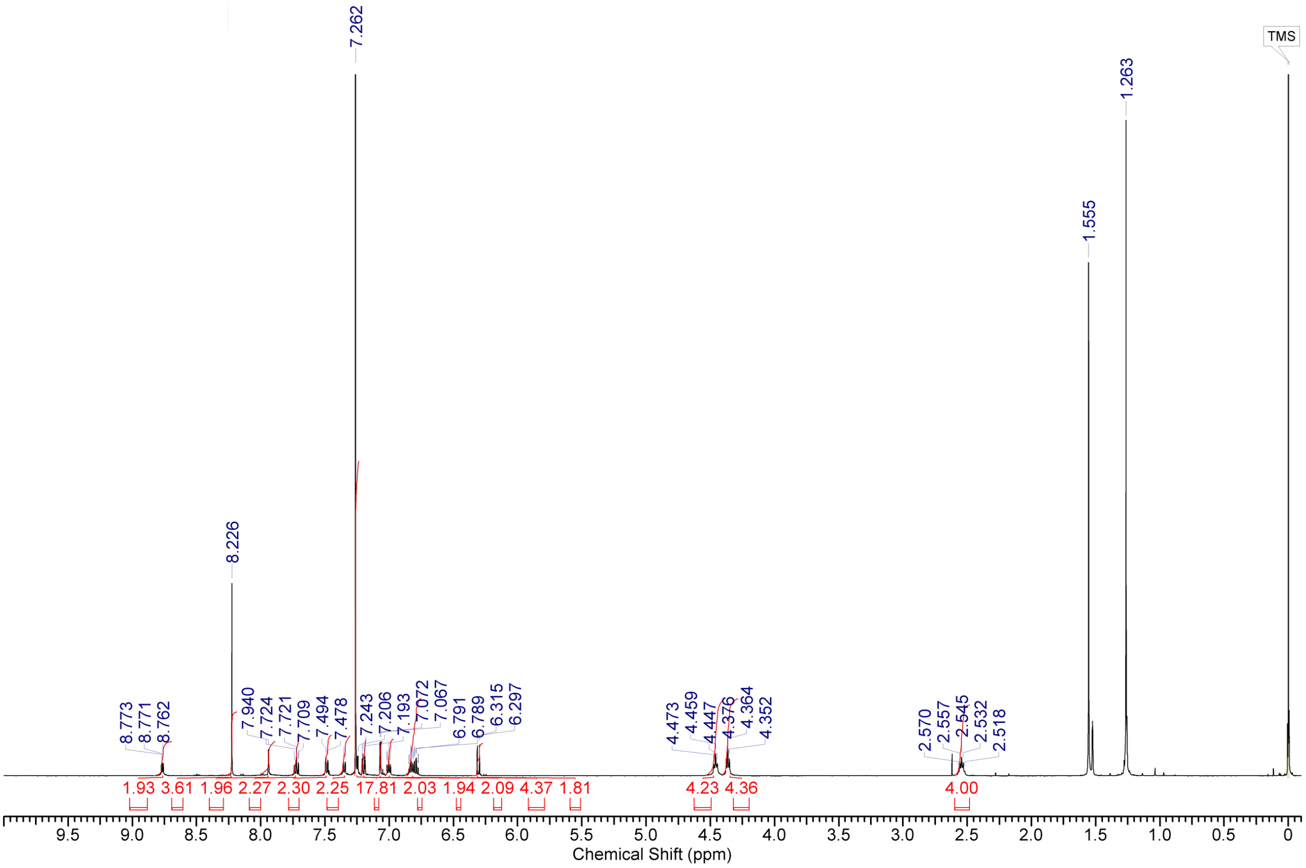

Figure S6. ^1H NMR spectra (500 MHz) of **2a** in $CDCl_3$.

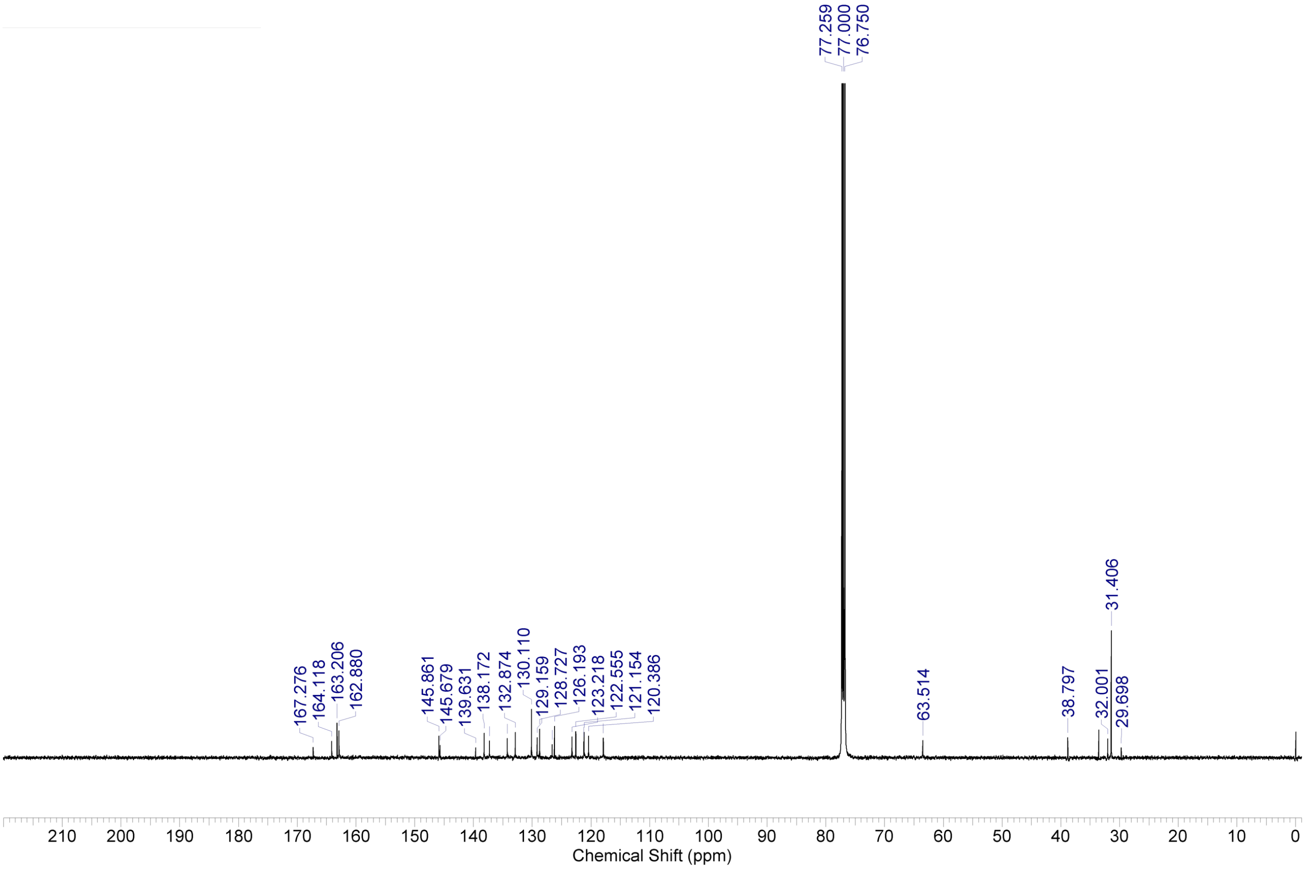

Figure S7. ^{13}C NMR spectra (125 MHz) of **2a** in $CDCl_3$.

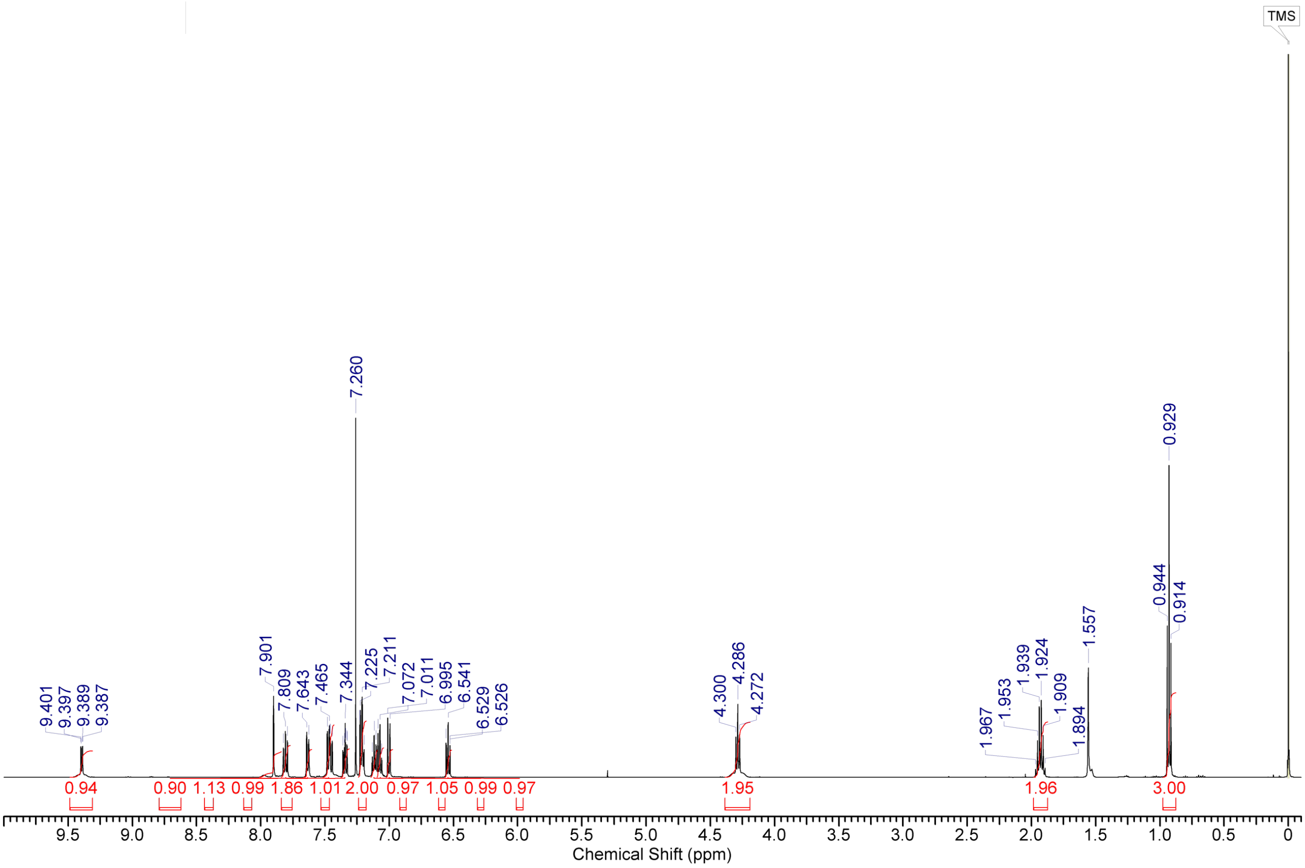

Figure S8. ^{1}H NMR spectra (500 MHz) of **3** in $CDCl_3$.

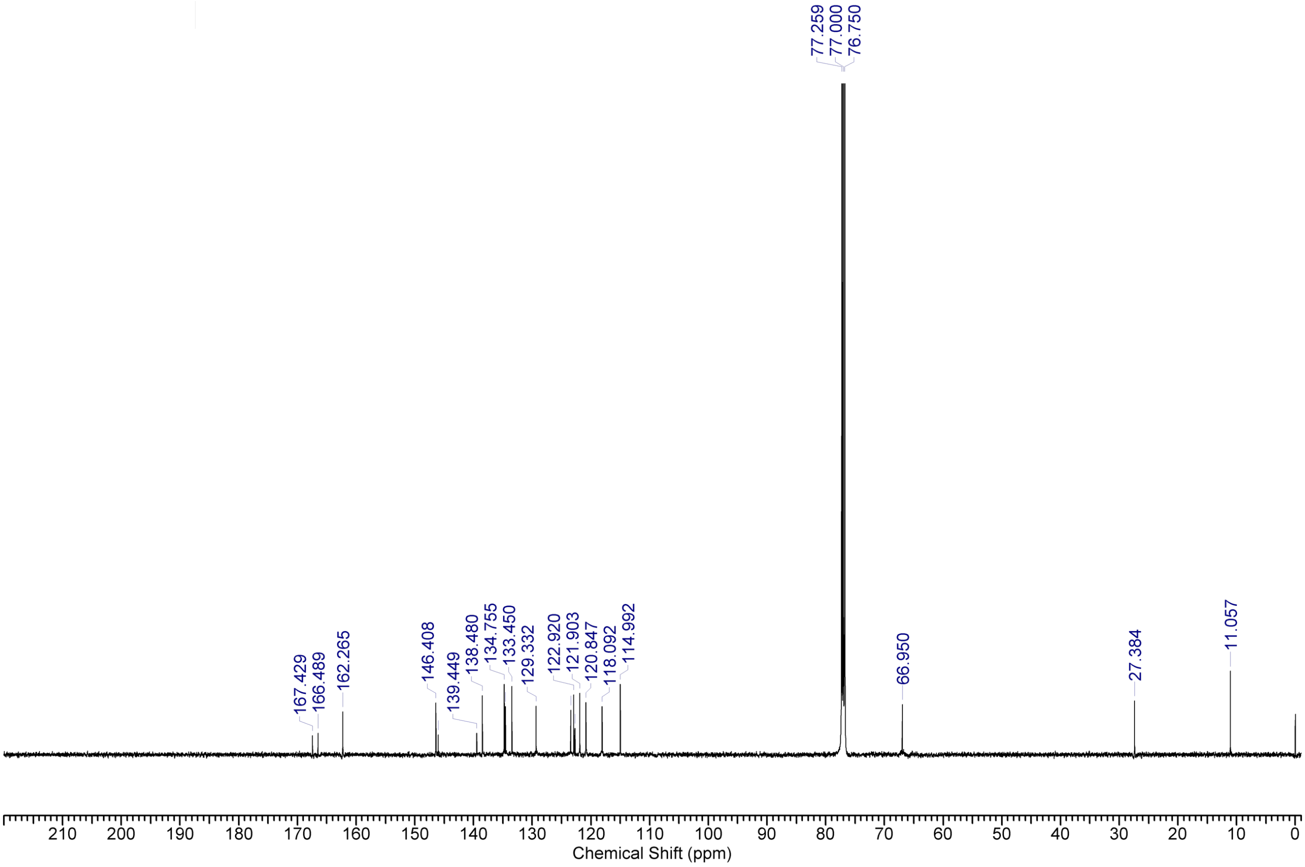

Figure S9. ^{13}C NMR spectra (125 MHz) of **3** in $CDCl_3$.

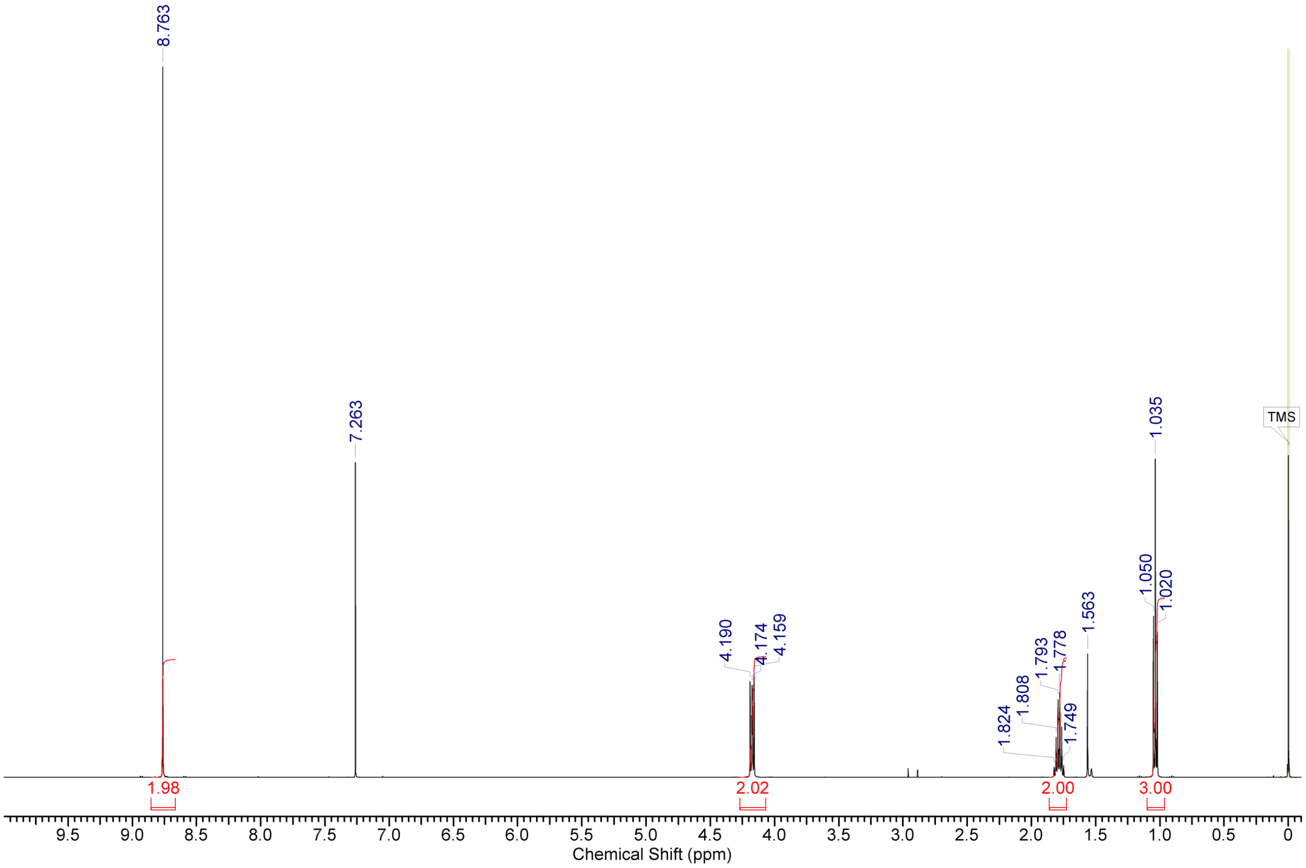

Figure S10. ^{1}H NMR spectra (500 MHz) of **4** in $CDCl_3$.

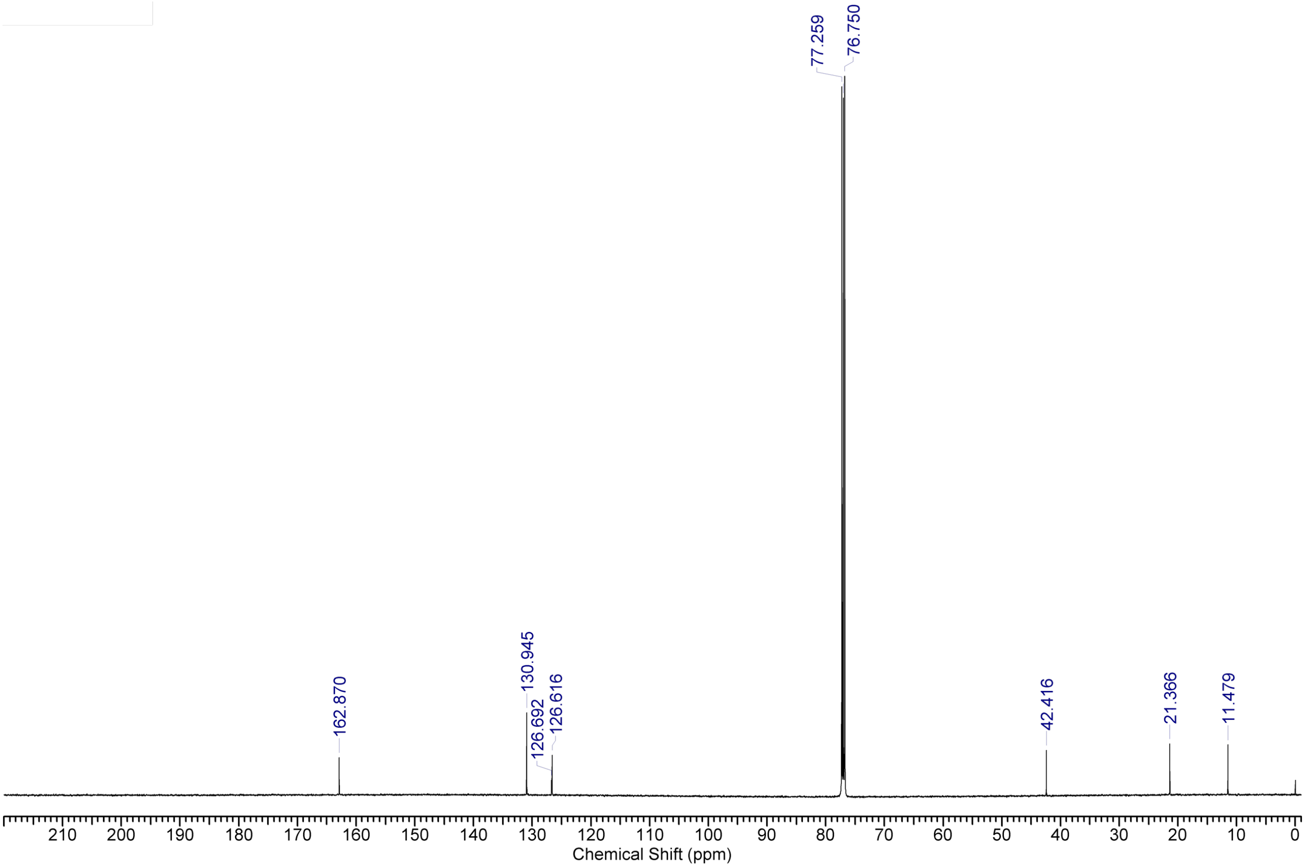

Figure S11. ^{13}C NMR spectra (125 MHz) of **4** in $CDCl_3$.
